# Perinatal Transmission of SARS-CoV-2 Infection and Its Clinical Attributes: A Single-Center Study From Western Uttar Pradesh

**DOI:** 10.7759/cureus.35824

**Published:** 2023-03-06

**Authors:** Shazmeen Imran, Rakesh Gupta, Ritu Sharma, Sujaya Mukhopadhyay, Sanju Yadav

**Affiliations:** 1 Pediatrics, Government Institute of Medical Sciences, Greater Noida, Gautam Buddha Nagar, IND; 2 Obstetrics and Gynecology, Government Institute of Medical Sciences, Greater Noida, Gautam Buddha Nagar, IND

**Keywords:** horizontal sars-cov-2 transmission, neonatal outcome in maternal covid-19, in utero sars-cov-2 exposure, perinatal covid-19 infection, rooming-in and covid-19, nicu stay in covid-19 infection, hypocalcemia in neonatal covid-19, clinical attributes, vertical covid-19 transmission, western up

## Abstract

Background

Globally, severe acute respiratory syndrome coronavirus 2 (SARS‑CoV‑2) has infected millions of people to date. The morbidity and mortality associated with SARS-CoV-2 are higher in diabetics than those with chronic kidney disease and in the elderly. In pregnant women, it causes an increased risk for preeclampsia/eclampsia, infections, intensive care unit (ICU) admission, maternal mortality, and preterm birth. In neonates, SARS‑CoV‑2 infection has been found to cause stillbirths, growth retardation, premature delivery, increased neonatal intensive care unit (NICU) admission, and need for oxygen support. The neonate can get infected by vertical or horizontal transmission. As most studies have focussed on transmission at the time of birth only, in this study, we explored both vertical and horizontal transmission along with the clinical attributes of those born to mothers with SARS‑CoV‑2 infection.

Methodology

A prospective observational study was conducted in the Department of Pediatrics of a tertiary care hospital over 12 months from October 2020 to October 2021. All reverse transcription-polymerase chain reaction (RT-PCR) SARS-CoV-2-positive pregnant females admitted to the facility during the study duration were included. The enrolled mothers were followed till delivery. The mothers and neonates were managed per standard guidelines. Delivery details and neonatal outcomes were recorded. Coronavirus disease 2019 sampling in newborn babies was done at birth (within 24 hours) using a nasopharyngeal swab sample for RTPCR along with cord blood for SARS-CoV-2 immunoglobulin M (IgM). Complete blood count, C-reactive protein, serum electrolytes, random blood sugar, and chest X-ray were obtained for all babies at birth and thereafter according to requirement. In those roomed in with their mother, RT-PCR was repeated at the time of discharge or if they became symptomatic.

Results

A total of 44 mother-neonate dyads were included in the study. Cord blood IgM for SARS‑CoV‑2 was negative for all neonates, while throat swab RT-PCR was positive for two (4.5%) neonates immediately after birth. Overall, 13.6% of the neonates were premature, 27.2% of the neonates had low birth weight (<2,500 g), and 6.8% had very low birth weight (<1,500 g). Among those admitted to the NICU, 18.2% had respiratory distress; 4.5% had fever, lethargy, and poor feeding; and hyperbilirubinemia requiring phototherapy was observed in 11.3% of the neonates. Moreover, 4.5% of the neonates had hypocalcemia on initial investigations. Mortality was seen in 2.2% (1/44) of the neonates. Rooming-in and breastfeeding were seen in 68.2% of the neonates. The horizontal transmission was seen in one (3.3%) roomed-in neonate.

Conclusions

Perinatal transmission of SARS‑CoV‑2 infection does occur but its rate is not significant. Furthermore, with proper infection prevention and control measures, the risk of perinatal transmission can be decreased. Breastfeeding and rooming-in do not increase infection transmission if the mother takes all precautions.

## Introduction

Globally, to date, severe acute respiratory syndrome coronavirus 2 (SARS‑CoV‑2) infection has infected 752,517,552 people with 6,804,491 deaths [1]. The morbidity and mortality associated with SARS-CoV-2 have been found to be higher in diabetics compared to those with chronic kidney disease (CKD) and in the elderly [2]. In pregnant women with SARS‑CoV‑2 infection, an increased risk for preeclampsia/eclampsia, infections, intensive care unit (ICU) admission, maternal mortality, and preterm birth has been seen. In a literature review, Mark et al. [3] identified 28% preterm birth and 57% cesarean section in women with coronavirus disease 2019 (COVID-19) who gave birth. In neonates, SARS-CoV-2 infection can lead to many adverse outcomes such as growth retardation, premature delivery, and neonatal intensive care unit (NICU) admission, though the incidence varies among different studies [4,5].

In a meta-analysis by Kyle et al. [5], it was seen that in the over 800 newborns reported in the literature, the incidence of vertical transmission was low. Similarly, in a systematic review by Yuan et al. [6], after an extensive literature search, they found a positivity rate of 3.28% (18/549). They concluded that there is no sufficient evidence to rule out the possibility of risk of vertical transmission. Vertical transmission of SARS-CoV-2 is rare, and it most commonly occurs in the third trimester of pregnancy [7]. It is likely that the fetus gets infected by encountering infected maternal fluids during labor [8].

Hosier et al. [9] and Pulinx et al. [10] reported that syncytiotrophoblast cells are mainly infected. These are the cells involved in the transfer of nutrients from the mother to the fetus. Hence, they can be the transferring agent. However, many studies have negated vertical transmission. Wu et al. [11] observed 30 neonates among 29 pregnant COVID-19-infected women and found that five were diagnosed with COVID-19 infection. They opined that intrauterine or intrapartum transmission is possible and requires further investigation. Facchetti et al. [7] screened for SARS-CoV-2 spike (S) protein expression in the placentas of 101 women who delivered between February 7 and May 15, 2020, many of whom tested positive. They found that SARS-CoV-2 S and N proteins were strongly expressed in the placenta of a COVID-19 pregnant woman whose newborns tested positive for SARS-CoV-2 infection and developed COVID-19 pneumonia after birth. They also found SARS-CoV-2 antigens, RNA, and/or other particles consistent with coronavirus in villous syncytiotrophoblast, endothelial cells, fibroblasts, maternal macrophages, Hofbauer cells, and fetal intravascular mononuclear cells. Thus, they opined that maternal-fetal transmission of SARS-CoV-2 is likely by circulating virus-infected fetal mononuclear cells. However, some researchers found that there is inefficient virus replication in placental tissues, and the immune system of the fetus and the angiotensin-converting enzyme 2 (ACE2) receptors are immature, thus inferring that vertical transmission of SARS-CoV-2 is rare [12].

Horizontal transmission in a neonate is considered if the initial negative reverse transcription-polymerase chain reaction (RT-PCR) test within the first 72 hours subsequently turns positive after 72 hours of birth irrespective of the mother’s SARS-CoV-2 status [13,14]. A few studies have examined the incidence of horizontal transmission and found that it was less than vertical (8% vertical and 1.5% horizontal) [15]. As most studies have only focused on transmission at the time of birth, in this study, we explored both vertical and horizontal transmission as well as the clinical attributes of those born to mothers with SARS-CoV-2 infection.

## Materials and methods

This prospective observational study was conducted in the Department of Pediatrics of a tertiary care hospital over 12 months from October 2020 to October 2021 after obtaining ethical permission (approval number: IEC/HR/2020/29).

Inclusion and exclusion criteria

This study included all RT-PCR SARS-CoV-2-positive pregnant females with gestational age ≥28 weeks who were admitted to the facility during the study duration, those who delivered a live baby in the institute, and those who consented to participate.

We excluded mothers who had preexisting medical illnesses such as hypertension, diabetes mellitus, and anemia; those who had a stillbirth; and pregnant patients without RT-PCR-confirmed SARS-CoV-2 infection.

The enrolled mothers were followed till delivery. The mothers and neonates were managed per standard guidelines followed by the obstetricians and pediatricians in the institute.

Apart from delivery details, neonatal outcomes including birth weight and gestation, neonatal symptoms at birth (fever, lethargy, respiratory symptoms, or intolerance to feeding), NICU admission, and neonatal mortality during the hospital stay were recorded. COVID-19 sampling in newborn babies was done at birth (or within 24 hours) by a nasopharyngeal swab sample for RTPCR along with cord blood for SARS-CoV-2 immunoglobulin M (IgM). Complete blood count (CBC), C-reactive protein (CRP), serum electrolytes (SEs), random blood sugar (RBS), and chest X-ray (CXR) were done in all babies at birth and thereafter per requirement. In addition, we recorded the duration of oxygen support, continuous positive airway pressure (CPAP) support, or mechanical ventilation.

Sick neonates needing hospitalization for any reason were managed in the NICU. Well neonates were either roomed in with the mother or handed over to the family depending upon their choice. In those roomed in with the mother, RT-PCR was repeated at the time of discharge or if they became symptomatic. Repeat throat swab RT-PCR of COVID-19-positive neonates was done after five days. The data were recorded on a predesigned proforma.

Statistical analysis

Continuous variables were presented as mean ± standard deviation (SD) or median with range, while categorical variables were presented as numbers and percentages (%). The Student’s t-test was used to compare the normally distributed data. We also used the chi-square test and Fisher’s exact test according to the requirement. P-values <0.05 were considered statistically significant. Regression models were used to assess risk factors. We used SPSS version 21.00 software (IBM Corp., Armonk, NY, USA) for statistical analysis.

## Results

During the study period, 52 SARS-CoV-2-positive mothers in their third trimester delivered at our center. Of them, two had stillbirths and were excluded. We approached the remaining 50 for participation. Six refused and 44 agreed to be part of the study. Hence, they were included and the characteristics of their neonates were recorded.

Cord blood IgM for SARS-CoV-2 was negative for all neonates, while throat swab RT-PCR was positive for two (4.5%; 2/44) neonates immediately after birth, and one (3.3%; 1/30) neonate became positive after rooming-in on day 10 of life. In neonates who were RT-PCR positive at birth, repeat RT-PCR was done on day five of life which turned out to be negative. RT-PCR of roomed-in neonates was repeated on day 15 of life, which was also negative.

In total, six (13.6%) neonates were delivered prematurely. Twelve (27.2%) neonates had low birth weight (LBW) (<2,500 g) and three (6.8%) had very low birth weight (VLBW) (<1,500 g). However, only four (9.1%) neonates were small for gestational age (SGA). Overall, 19 (43.2%) neonates were delivered by cesarean section. Fetal distress and meconium-stained liquor were noted in five (11.3%) deliveries, and one (2.2%) neonate required resuscitation at birth.

In the NICU, 19 (43.1%) neonates were admitted, among whom 15 (34.1%) were symptomatic, eight (18.2%) had respiratory distress; two (4.5%) had fever, lethargy, and poor feeding; hyperbilirubinemia requiring phototherapy was observed in five (11.3%) neonates; two (4.5%) neonates were found to be hypoglycemic at birth; and two (4.5%) neonates had hypocalcemia on initial investigations. The median duration of stay was two days (interquartile range (IQR) = 1-5), varying between a few hours to 15 days. One (2.2%; 1/44) of the admitted neonates expired on day 12 due to prematurity/hyaline membrane disease/sepsis and its complications. The neonate was SARS-CoV-2 negative.

Finally, rooming-in and breastfeeding were seen in 30 (68.2%) neonates. At the time of discharge, one (2.2%) roomed-in neonate tested positive for COVID-19 RT-PCR (Table 1).

**Table 1:** Baseline characteristics. LSCS = lower segment cesarean section; NVD = normal vaginal delivery; AGA = appropriate for gestational age; SGA = small for gestational age; LGA = large for gestational age; LBW = low birth weight; VLBW = very low birth weight; NICU = neonatal intensive care unit

Parameter	Number (%), N = 44
Total patients	44
COVID-19-positive newborns at birth	2 (4.5)
COVID-19-positive newborns after rooming in (N = 30)	1 (3.3)
COVID-19-negative newborns	41 (93.2)
Average gestational age (weeks) (mean ± SD)	38 ± 2.5 (30–42 weeks 3 days)
Average weight (g) (mean ± SD)	2,625 ± 522 (1,500–4,000)
LSCS	19 (43.2)
NVD	25 (56.8)
Term	38 (86.4)
Preterm	6 (13.6)
Male	29 (65.9)
Female	15 (34.1)
AGA	39 (88.6)
LGA	1 (2.3)
SGA	4 (9.1)
LBW	12 (27.2)
VLBW	3 (6.8)
Sepsis	4 (9.1)
Hypocalcemia	2 (4.5)
Resp distress and oxygen support	8 (18.2)
Rooming in and breastfeeding	30 (68.2)
NICU stay	19 (43.2)
Mortality	1 (2.2) SARS-CoV-2 negative neonate

On comparing COVID-19-positive and COVID-19-negative neonates, no significant difference was observed in terms of sepsis, respiratory distress, rooming-in, and breastfeeding, and no significant difference was seen except in the incidence of hypocalcemia (p = 0.01) (Table 2).

**Table 2:** Difference between SARS-CoV-2-positive and negative neonates. **: Fisher’s exact test was used. LSCS = lower segment cesarean section; NVD = normal vaginal delivery; AGA = appropriate for gestational age; SGA = small for gestational age; LGA = large for gestational age; LBW = low birth weight; VLBW = very low birth weight; NICU = neonatal intensive care unit

Parameter	Total, N = 44 Number (%)	SARS-CoV-2 positive, N = 3 Number (%)	SARS-CoV-2 negative, N = 41 Number (%)	^**^P-value
LSCS	19 (43.2)	1 (33.3)	18 (43.9)	0.72
NVD	25 (56.8)	2 (66.7)	23 (56.1)	
Term	38 (86.4)	3 (100)	35 (85.4)	0.47
Preterm	6 (13.6)	0 (0)	6 (14.6)	
Male	29 (65.9)	2 (66.7)	27 (65.8)	0.97
Female	15 (34.1)	1 (33.3)	14 (34.1)	
AGA	39 (88.6)	3 (100)	36 (87.8)	0.81
LGA	1 (2.2)	0 (0)	1 (2.4)	
SGA	4 (9.1)	0 (0)	4 (9.7)	
Sepsis	4 (9.1)	0 (0)	4 (9.7)	0.57
Hypocalcemia	2 (4.5)	1 (33.3)	1 (2.4)	0.01
Respiratory distress and oxygen support	8 (18.2)	1 (33.3)	7 (17.1)	0.48
Rooming-in and breastfeeding	30 (68.2)	3 (100)	27 (65.9)	0.220

## Discussion

Infection in neonates can be transmitted through the placenta (in utero transmission), during labor by aspiration of amniotic fluid and fetoplacental bleed, and late neonatal infection by breastfeeding or acquired from an infected caregiver/mother.

In our cohort, the rate of vertical transmission judged by positive throat swab RT-PCR at birth was 4.5% and that of horizontal transmission after rooming-in was 3.3%. Cord blood IgM was negative for all neonates. Rooming-in and breastfeeding were seen in 68.2% of neonates in our study. Different studies by various researchers have found the rate of vertical transmission to range between 2% and 10.7% [11,14-16]. While the rate of horizontal transmission was 1.5% in a meta-analysis by Moore et al. [14]. Raschetti et al. [17] performed a meta-analysis of 176 published cases of neonatal SARS-CoV-2 infections defined by at least one positive nasopharyngeal swab and/or the presence of specific IgM. The study reported horizontal transmission in 70% of neonatal COVID-19 infections. The study found that environmental factors such as rooming-in and not completely adhering to infection prevention and control (IPC) practices were the main reasons. Proving the same finding, a study by Salvatore et al. [18] found no perinatal transmission among 116 SARS-CoV-2-positive pregnant women with rooming-in and breastfeeding when IPC practices such as hygiene precautions, maternal masking, and parental education were followed. Anand et al. [16] reported the horizontal transmission rate to be 7.8% (3/38) in mother-infant dyads following rooming-in and breastfeeding. Hence, it can be safely judged that adhering to IPC practices is crucial in the fight against COVID-19.

The incidence of prematurity in our study was 13.6%, and the median gestational age was 38.85 weeks. None of the positive neonates were preterm. In general, the rate of preterm delivery in India is 18.8% while studies from India have reported a variable incidence of prematurity during the COVID-19 pandemic between 4.78% and 28.93% [19]. A study by Salvatore et al. [18] from New York City found that 17% (14/82) of neonates were preterm, and the median gestational age was 38 weeks (range = 27-41). The main reasons were maternal COVID-19 leading to hypoxemia, fetal distress, and birth asphyxia or meconium-stained amniotic fluid [20-22].

In the present study, 27.2% of neonates weighed <2,500 g and 6.8% weighed <1,500 g. Hence, in total, 34% of the neonates had a weight lower than average which was comparable to reports from other centers in the country (19.8-50%) [23,24]. In a meta-analysis by Panda et al., the pooled rate of LBW in India across 14 studies was 28.6% [19].

In our study, 43.1% of neonates required NICU care. The main reasons for admission were respiratory distress requiring oxygen support followed by sepsis (18.2% and 9.1%, respectively). The average duration of stay was two days, varying between a few hours to 15 days. Other studies report a variable percentage of NICU admission between 23.1% and 27.1% [15,25]. In our center, the NICU stay was higher than that seen in other centers. One of the reasons was that about 9% of neonates were admitted as their mothers were sick and caretakers were not available to care for them at home.

In other studies, the need for oxygen support varied between 1.0% and 15.7% [3,26-29]. Our center witnessed a higher oxygen requirement in neonates born to COVID-19-positive mothers (18.2%). One neonate required mechanical ventilation while four needed CPAP support. The rest improved with oxygen support by nasal prongs. In a study by Norman et al., the need for CPAP and mechanical ventilation was higher in neonates born to mothers with SARS-CoV-2 infection (4.9% vs. 3.8%; odds ratio (OR) = 1.32; 95% confidence interval (CI) = 1.06-1.64; 1.6% vs. 0.5%; OR = 3.51; 95% CI = 1.856.65) [3]. The mean duration of stay found in another study was comparable to ours at 2.5 days (IQR = 2.5-7) [21].

The mortality rate observed in our study was 2.2%. The reason was prematurity, hyaline membrane disease, sepsis, and other complications. In a multinational retrospective cohort study conducted in 73 centers from 22 countries from February 1, 2020, to April 30, 2020, by Saccone et al. [25], 2.0% of neonatal deaths were reported.

In the present study, we found a significant difference in the incidence of hypocalcemia among COVID-19-positive and negative neonates (p = 0.01). This can be because of inequality in the sample size of the two groups. No significant difference was seen in the rest of the parameters between COVID-19-positive and negative neonates. Though hypocalcemia is a known prognostic marker of the severity of COVID-19 infection in adults [30], its relationship with neonatal COVID-19 is not well documented. It can be researched further in the coming years.

We have described the rate of vertical and horizontal transmission among the cohort along with the clinical attributes of the three SARS-CoV-2-positive neonates (6.8% total). None had severe illness and were discharged.

Strengths and limitations

Few studies have compared vertical and horizontal transmission and none from this part of the country, which is a strength of this study. However, we could not enroll controls as our institute was a dedicated COVID-19 facility at the time and we were catering to only COVID-19-positive patients.

## Conclusions

Having seen the wrath of COVID-19 infection worldwide, the panic is obvious when a neonate is born to a SARS-CoV-2-infected mother. This study concludes that perinatal transmission of SARS-CoV-2 infection does occur but the rate is not significant. Further, with proper IPC measures, the risk of perinatal transmission can be decreased. Breastfeeding and rooming-in do not increase infection transmission if the mother takes all precautions. Hence, these practices should be encouraged. There should not be any need for panic in further waves of infection.
